# Cataloging the potential functional diversity of *Cacna1e* splice variants using long-read sequencing

**DOI:** 10.1186/s12864-025-11887-1

**Published:** 2025-09-26

**Authors:** Shamsuddin A. Bhuiyan, John R. Tyson, Manuel Belmadani, Jordan Sicherman, Terrance P. Snutch, Paul Pavlidis

**Affiliations:** 1 Michael Smith Laboratories, University of British Columbia, Vancouver, BC V6T 1Z4 Canada; 2 Department of Psychiatry, University of British Columbia, Vancouver, BC V6T 1Z4 Canada; 3 Bioinformatics Training Program, University of British Columbia, Vancouver, BC V6T 1Z4 Canada; 4 Djavad Mowafaghian Centre for Brain Health, University of British Columbia, Vancouver, BC V6T 1Z4 Canada

**Keywords:** Calcium channels, Nanopore sequencing, Alternative splicing, Noisy splicing model

## Abstract

**Background:**

The degree to which alternative RNA splicing influences the function and structure of voltage gated calcium channel (VGCC) splice variants is poorly understood. Here we used long-read RNA-sequencing to catalog rat *Cacna1e* (Cav2.3) splice variants, and computationally prioritize which are likely to impact channel function.

**Result:**

We sequenced *Cacna1e* transcripts from rat thalamus using Oxford Nanopore sequencing yielding the structure of 2,110 *Cacna1e* splice variants. Of these, up to 154 had the potential encode for a functional channel based on predicted amino acid sequences. Our analysis revealed a total of 31 cassette splicing events (in various combinations) potentially affecting channel function, with three cassette exons appreciably expressed and conserved.

**Conclusion:**

Our work both provides the first long-read sequencing of *Cacna1e* and the first computational evaluation of *Cacna1e* splice variants for future follow-up. This overall strategy to provide the field with prioritized transcripts will improve our understanding of *Cacna1e* function, its role in disease pathophysiology, and serve as a general approach to evaluate splice variant function across multiple ion channel types.

**Supplementary Information:**

The online version contains supplementary material available at 10.1186/s12864-025-11887-1.

## Background

Voltage-gated calcium channels (VGCCs) play crucial roles in regulating calcium ion influx into neurons. Mutations in the pore-forming α_1_-subunit genes that encode VGCCs (CACNA1x, also termed Cavs) are linked to neurological disorders such as epilepsy, migraine and schizophrenia [1]. Despite their importance, our understanding of VGCCs remains incomplete with respect to their extensive alternative splicing. An important contribution will be to define the complete splicing repertoire of each VGCC subtype. Here, we describe our work to profile the distinct splice variants of the rat *Cacna1e* gene using long-read RNA-sequencing.

*Cacna1e* encodes the α_1_-subunit of the Cav2.3 channel [2, 42, 52]. Cav2.3 currents uniquely exhibit biophysical and pharmacological characteristics representative of both high-voltage-activated and low-voltage-activated VGCCs. *Cacna1e* knock-out in rodents resulted in decreased calcium currents in hippocampal pyramidal cells, increased sensitivity to inflammatory pain, and resistance to drug-induced seizures [2–7]. *Canca1e* gain-of-function mutations have been associated with epileptic encephalopathy, macrocephaly, and dyskinesia [7–10].

The large *Cacna1e* gene contains at least 36 exons, and previous literature reports multiple possible splice variants [3], albeit with most having an unclear impact on channel function. We note that “alternative” splice variants have the potential to either not affect function or be transcriptional noise that does not produce a functional product at all [11–14]. For example, there are constraints on *Cacna1e* transcripts to form functional calcium channels: the encoded α1-subunit must be at least 2000 amino acids long and contain four pore-forming domains [15] with mutations in any of the pore-forming domains possibly resulting in channel dysfunction [16]. The multiple splice variants identified to date for *Cacna1e* generally possess similar exons encoding for the conserved pore-forming domains; however they vary in their exons located in between the pore-forming domains (the linker regions) and carboxyl terminal regions [17, 18].

When examining eight human *CACNA1E* “alternative” splice variants, Pereverzev and colleagues found that different variants encode for channels with distinct inactivation and recovery time courses [19]. Additionally, Klockner and colleagues noted distinct binding affinities among regulatory proteins and *CACNA1E* splice variants [20]. Given that CAV2.3 antagonists (e.g. Topiramate) are used to treat seizures and different splice variants have different pharmacological sensitivities, understanding the role of *CACNA1E* splice variation can potentially aid in drug development efforts [17, 21]. In these regards, establishing the role of *CACNA1E* splice variants in relation to neuronal activity first requires an accurate catalog of *CACNA1E* splice variants.

A technical limitation of previous studies cataloging VGCC alternative splicing is the use of short-read sequencing (~ 150–175 bp) [22, 23], which is unable to resolve the relationship between an individual short read and full length transcript structure. An alternative to short-read sequencing, long-read nanopore-derived RNA-seq data using Oxford Nanopore Technology (ONT) technology provides the opportunity to more accurately define the splice variant repertoire [24]. Previously, Clark and colleagues used MinION sequencing to establish *CACNA1C* transcriptional complexity in the human brain [24]. They reported that only a trivial minority of the *CACNA1C* splice variants they identified had been previously included in transcript databases (e.g. Ensembl, Refseq) and speculated that these additional variants contribute to the functional diversity of *CACNA1C* in different brain regions. However, similar data sets are lacking for other VGCCs.

The lack of a comprehensive *CACNA1E* splice variant catalog likely impacts the various genomic databases that report splice variants such as Ensembl, and highlights the previously noted disconnect between computational tools and the experimental literature [12, 25]. Bhuiyan and colleagues previously reported the failure to identify splice variants in Ensembl for about a third of genes with literature evidence for functionally distinct splice isoforms (FDSIs) [12]. This disconnect impacts the ability to computationally evaluate alternative splicing in genes with complex splicing patterns.

In this study, we characterized the splicing profile for *Cacna1e* using ONT RNA-seq data from the rat thalamus. We identified the structures and splice junctions for 2,110 *Cacna1e* splice variants. Using computational annotations, we established a putative set of 154 functional *Cacna1e* transcript variants. In doing so, we have added to the potential functional diversity of the Cav2.3 calcium channel while reporting a more accurate characterization of splice variant structures than previously available. This improved transcript catalog is predicted to be more widely applicable in aiding computational tools and providing experimentalists potentially interesting splice variants for further investigation.

## Methods

### Targeted amplification of *Cacna1e* GAERS and NEC rats

We chose to investigate *Cacna1e* splicing in P10 and P90 GAERS and NEC (Non-Epileptic Control) rats due to the extensive history of using these animals to model seizures [26–28]. More specifically, much of the GAERS literature has focused on linking thalamus dysregulation to seizures. This study served as a proof-of-concept for establishing the tools and methodology to catalog and predict splice variants within a complex RNA pool in the rat thalamus. Consequently, it was not designed to account for potential sex differences. The P10 and P90 developmental stages were chosen due to well-documented transcriptional changes between these time points, which are relevant to the development of absence seizures in GAERS compared to NEC [29, 30]. All animal procedures were performed in accordance with the University of British Columbia Animal Care Committee regulations and in accordance with animal care regulation and policies of the Canadian Council on Animal Care. GAERs and NEC rats were generously gifted to TPS by Terrance O’Brien from the University of Melbourne and the rat colonies have been maintained at the University of British Columbia. Rats had unlimited access to a diet of LabDiet 5001 Rodent Diet and water. Prior to surgical extraction of the thalamus, rats were anesthetized using isoflurane and euthanized via decapitation. Dissected tissue was then flash-frozen in liquid nitrogen and stored at −80 °C.

We performed targeted amplification of *Cacna1e* transcripts from the dissected thalamus of male GAERS and NEC rats. Total RNA was extracted from the thalamus of 2 GAERS (10 day and 90 days postnatal) and 2 NEC (10 and 90 days postnatal) male rats using a MagMax Kit (Ambion) and full length cDNAs generated using SuperScriptII with oligo-dT priming (Invitrogen) as per the Oxford Nanopore Technologies protocol for the cDNA-PCR Sequencing kit. These samples will be referred to as GAERS10, GAERS90, NEC10 and NEC90. Gene-specific amplicons for the *Cacna1e* gene (Transcript: XM_039091007.2; Forward: ATGGCTCGCTTCGGGGAG; Reverse: CTAGCACTTATCGTCTTCTTC) were generated using PCR with the Enlongase enzyme (Invitrogen). PCR products were then agarose gel-purified using a gel extraction kit (QIAGEN) and DNA eluted in TE and stored at −20 °C until DNA sequencing.

### ONT MinION sequencing of amplicons

The generated *Cacna1e* gene specific amplicons were processed as per the Oxford Nanopore Technologies (ONT) SQK-LSK108 adapter Ligation procedure. In brief, DNA amplicon molecules were treated to generate A-tailed molecules allowing the ligation of ONT specific adapter onto the amplicon ends. These adapted molecules were then run on the ONT MinION device and signal data captured for base calling over a 48 h period. Sequence data was generated from the raw captured data using ONT specific software (Guppy 3.4) on a GPU enabled desktop PC. Raw sequence data are available in GEO under accession GSE295903.

### Short-read sequencing of genes and alignment

While third-generation sequencing effectively captures full-length transcripts and complex splicing patterns, it has a higher error rate than short-read sequencing. To improve accuracy, we used short-read RNA-seq to correct potential errors in long-read data, particularly in splice site detection, similar to studies like Tang et al. [31] and Glinos et al. [32].

The generated gene-specific amplicons were short-read sequenced at the BC Genome Sciences Center and data was provided back in SOLEXA format. The SOLEXA format was designed for use with Solexa/Illumina sequencing platforms and represents each sequence read alongside a quality score that quantifies the confidence in each base call. These quality scores, unique to the SOLEXA format, are calculated as log-odds ratios and encoded using ASCII characters. Raw sequence data are available in GEO under accession GSE295903.

We reprocessed the rat transcriptomic SOLEXA data. Since these reads were in SOLEXA format, we converted them into FASTQ format in order to run them through our short-read RNA-seq pipeline.

The rat transcriptome reference was prepared using the “rsem-prepare-reference” script provided by the software package"RNA-seq by expectation–maximization"in RSEM [33]. The assembly version used was Ensembl Rnor6.0, obtained through Illumina for the iGenomes collection. (https://support.illumina.com/sequencing/sequencing_software/igenome.html). Short-reads were processed as single-end (no mate pairs) and aligned using the STAR aligner (Dobin et al., 2013) version 2.4.0 h. We provided mapped reads as input to the quantification scripts from RSEM v1.2.31. Default parameters were used (except for parallel processing and logging related options). The count quantification matrix of splice junctions (SJ.tab.out) was used for downstream FLAIR analysis.

### Determining splice variants using long read RNA-seq data and FLAIR

For the purposes of identifying novel splice variants found in the rat thalamus, all four samples were combined for downstream analysis. We aligned our long-reads for each sample to the Ensembl rat genome (rn6) using FLAIR [34] and minimap2 [35]. To address the higher error rate of Nanopore reads, we incorporated splice junctions identified from Illumina short-read data, allowing FLAIR to correct the reads in each alignment file. In short, this means that any novel splice junctions are merged to an existing splice junction if the novel junction is within a 10-nucleotide window of the existing junction. The 10-base parameter and the merging of splice junctions are default settings of the FLAIR program, which have been validated and employed in other studies, such as the GTEx long-read RNA-seq study (Glinos et al., 2022). Next, FLAIR collapsed any reads having the same transcription start site and same splice junctions across all samples into a single splice variant. FLAIR removed any splice variants without support from at least 3 reads. This step produced the final transcript structure for each splice variant. Finally, FLAIR quantifies the total number of reads assigned to each transcript. Note that at the quantification step, reads are reassigned to the final transcript structure produced during the collapse step. As a result, ambiguous reads can be reassigned to a different transcript and as a result, transcripts with Limited reads that are ambiguous can be reassigned to different transcripts between the collapse and the quantification step, resulting in splice variants without quantified reads. As a result, 2/2110 *Cacna1e* splice variants did not have any expression value.

### Definition of functional *Cacna1e* splice variant

We defined a gene with functionally distinct splice isoforms (FDSIs) as a gene with two or more splice isoforms that are necessary for the gene’s overall function. Under this definition, each individual isoform must cause a change in phenotype after experimental depletion [12]. For the purposes of computationally characterizing a candidate FDSI from *Cacna1e* splice variants, we defined a candidate *Cacna1e* FDSI as “a splice variant evolving under selection (conservation) with all 4 pore-forming domains necessary for calcium passage into the cell”. The requirement that the splice variant possess all 4 pore-forming domains implicitly means that the splice variant must have an open reading frame (ORF) of at least 6000 bp (2000 amino acids). Conserved splice variants indicate that the splice variant is necessary for reproductive success and sequence conservation acts as a proxy for functional importance.

### *Cacna1e* splice variant annotations

Our annotation pipeline is available on https://github.com/PavlidisLab/long_read_sequencing_Cacna1e.

We subsetted the 6,252 splice variants from FLAIR to 2,110 *Cacna1e* splice variants and then reduced all splice variants into their exons and identified exons shared between splice variants. Further, we annotated any exon that was not entirely found in Ensembl as novel. We then annotated all splice variants with the number of MinION reads which support it (Additional File 2).

As many transcripts are expressed at low levels, we consider genes with multiple “appreciably” expressed splice variants as stronger candidates for having FDSIs [36]. To prioritize *Cacna1e* appreciably expressed splice variants, we first ranked each variant according to its total expression across all samples. The variant with the highest expression was assigned a rank of ‘1,’ the second highest was given a rank of ‘2,’ and this ranking process continued for all identified splice variants. Next, we calculated the expression ratio of each splice variant relative to the rank 1 variant (the most highly expressed). This ratio was obtained by dividing the expression level of a given splice variant by the expression level of the rank 1 variant. For prioritization, we focused on splice variants with expression levels that were at least 50% of the rank 1 variant, corresponding to an expression ratio of less than 2. The threshold of an expression ratio of 2 was selected based on the approach described by Ezkurdia et al., 2015. Additionally, we calculated expression ratios using both raw expression values and TPM-normalized values to ensure robustness in our analysis.

We annotated all splice variants and their individual exons with their average PhastCons [37] and PhyloP [38] basewise scores from a 20 vertebrate species alignment, downloaded from the UCSC genome browser for build rn6 [39]. The average score for each exon was calculated using Kentutils. To determine if there was selection pressure upon the splice sites, we also annotated the average PhyloP conservation of the two intronic bases next to the 5’ side of the exon and the two intronic bases next to the 3’ side of the exon.

Using TransDecoder (https://github.com/TransDecoder/TransDecoder) we predicted translation products of the 2,110 splice variants from *Cacna1e* for any ORFs larger than 2,000 amino acids with a start codon, regardless of whether the ORF contains a stop codon. We then queried the Conserved Domain Database (CDD) with the translated sequences and annotated each splice variant with the protein domain hits the database returned [40].

We then performed homology searches against the GenBank nr database [41] on all exons using NCBI’s tBLASTn [42]. First, we extracted the sequences for all alternatively spliced exons. Each alternatively spliced exon’s sequence was concatenated with the sequences of its flanking exons. The set of three exonic sequences was BLAST-ed against the human, mouse, rat, zebrafish, fugu, coelacanth, lamprey and spotted gar data in the nr database. BLAST results were next filtered using an e-value threshold of 0.0001, gap threshold of less than 30%, query coverage threshold of 80% or more, and a percent identity threshold of 30% or more.

Our decision to use BLAST for analyzing exon conservation was inspired by prior work leveraging sequence homology and proteomics to assess splice variant conservation [14], and the construction of the CHESS database [25]. However, we acknowledge that integrating expression data provides an important complementary approach to refine BLAST-based analyses and distinguish biologically meaningful splice variants from potential noise. Therefore, we also compared splicing events involving prioritized exons to those identified in the GTEx long-read RNA-seq database [32].

### Splice site prediction using MaxEntScan

To assess the strength of splice donor and acceptor sites in *Cacna1e* transcripts, we used a computational approach based on MaxEntScan. Splice site prediction was performed on sequences extracted from Ensembl-annotated *Cacna1e* transcripts and our long-read RNA-seq data using the MaxEntScan 5′ and 3′ models, which evaluate the likelihood of splice site recognition based on empirical sequence motifs.

Using MaxEntScanPy, we used a custom Python script to identify canonical donor (GT) and acceptor (AG) splice sites within the sequences. The script first loads the MaxEntScan scoring matrices for donor and acceptor sites. It then reads input sequences from a FASTA file and searches for putative splice sites based on sequence motifs. Donor sites were identified as GT dinucleotides flanked by three upstream and six downstream bases (9-mers), while acceptor sites were defined as AG dinucleotides preceded by 20 upstream and three downstream bases (23-mers).

For each identified site, the script applied MaxEntScan’s score5 and score3 functions to calculate donor and acceptor strengths, respectively. The resulting scores, along with sequence ID, genomic position, site type, and sequence context, were written to an output file for further analysis.

This computational analysis allowed for direct comparison of splicing potential between Ensembl-annotated *Cacna1e* transcripts and our experimentally derived isoforms, providing insights into differences in splicing efficiency that may underlie transcript diversity.

### Visualization tool

We developed a visualization tool using R that uses three input files: a BED-formatted file of all splice variants (FLAIR output), a tab-delimited CDD output file, and a CSV formatted file of each splice variant’s expression. It parses exon start and size information to calculate chromosomal coordinates, normalizes isoform names across all inputs, and identifies preserved exon structures across splice variants of interest. The function also flags overlaps with predicted open reading frames (ORFs) and annotated protein domains. The visualization tool calculates, based on chromosomal position and exon sizes, the set of similarly annotated exons that are preserved between all splice variants of interest, and draws the result as a stack of aligned exon patterns. Since introns were disproportionately large as compared to the exons present in Cacna1e splice variants, drawing lengths of introns were made arbitrary. The tool and example inputs are available online at https://github.com/PavlidisLab/long_read_sequencing_Cacna1e.

## Results

We profiled the *Cacna1e* transcriptome using RNA isolated from the rat thalamus (*N* = 4 animals) for targeted MinION cDNA sequencing (see Methods). Our analysis encompassed 4,060,847 reads and initially yielded a raw data set of 2,110 potential splice variants (Tables 1 and 2; Additional File 1 and Additional File 2). The goal of our analysis was to identify which of these detected splice variants could likely form functional calcium channels and which are most likely to contribute to *Cacna1e*’s functional diversity.

**Table 1:** Summary of targeted MinION RNA-seq data

	**NEC 10**	**NEC 90**	**GAERS 10**	**GAERS 90**
# Reads	3,514,669	4,958,824	3,255,180	6,127,231
# Bases	20,259,569,128	23,582,737,080	19,092,042,421	31,856,992,171
Unmapped reads	31,356	31,356	17,311	5,773
Mean length of reads	5,764	4,756	5,865	5,199
Median length of reads	7,952	8,948	8,134	7,902
Number of reads FLAIR assigned to splice variants	3,494,983	4,881,246	3,243,064	6,076,453
Number of reads removed	19,686	77,578	12,116	50,778
Mean read quality	10.50	9.80	9.90	9.90
Median read quality	10.80	9.90	10.20	9.70

All four samples were sequenced with gene specific PCR primers. Column headings: NEC 10 – Non-epileptic control at 10 days, NEC 90 – Non-epileptic control at 90 days, GAERS 10—Genetic Absence Epilepsy Rat from Strasbourg at 10 days, GAERS 90—Genetic Absence Epilepsy Rat from Strasbourg at 90 days

**Table 2:** Summary of short-read RNA-seq data

	**Cacna1e—Thalamus**
# Reads	17,855,904
# Bases	94,791,340,800
Mapped reads	4,060,847

### Detection of candidate *Cacna1e* splice variants

Data were initially filtered for reads that mapped to *Cacna1e*, yielding an initial pool of 2,110 variants for *Cacna1e* that passed quality control (see Methods). Approximately 53% of the reads were at least 6,000 nucleotides, while median length of transcripts was 5,725 nucleotides. The number of reads that mapped to the same splice variant was used as a proxy for the splice variant expression level. We observed a positive correlation (Spearman’s *r* = 0.59) between splice variant size and the number of supporting reads per splice variant.

**Fig. 1:**
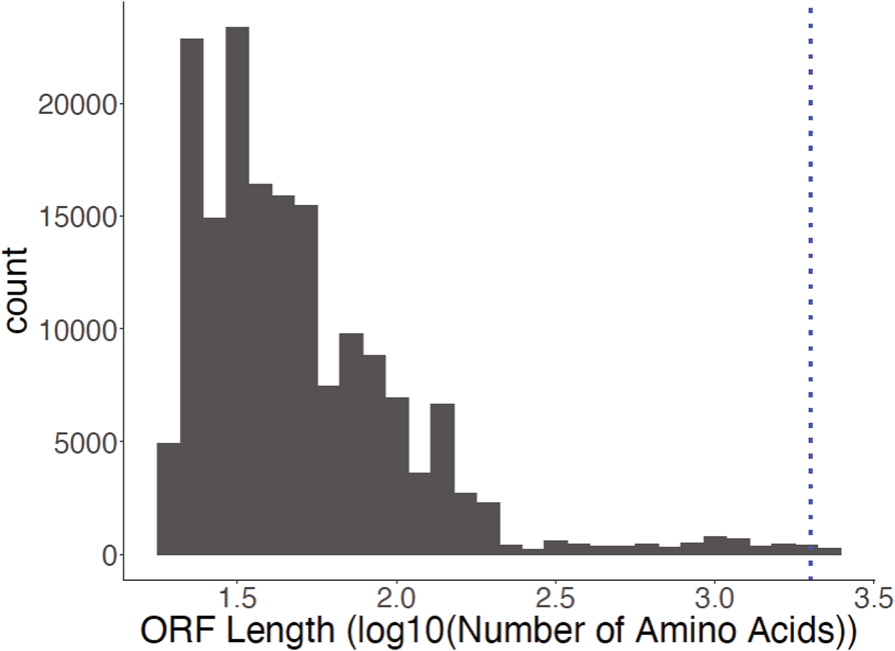
The majority of detected splice variants did not contain an open reading frame (ORF) of at least 2,000 amino acids. ORFs were defined as containing a start codon, but not necessarily a stop codon. The X-axis shows the distribution of log10(ORF) lengths. The dashed blue line indicates the minimum size expected for a functional channel

Once we identified the number of *Cacna1e* splice variants, we then determined how many of the splice variants had the translational potential for a functional α1-subunit (Table 3, Fig. 1). Of the 2,110 sequences passing quality control, 238 (11.3%) *Cacna1e* splice variants translated to a protein longer than 2,000 amino acids (Transdecoder predicted amino acid sequences in Additional File 3). Further, only based upon protein domain annotations from the Conserved Domain Database (CDD), 154 of the 238 (64.7%) *Cacna1e* splice variants had an ORF longer than 2000 amino acids that contained sequences encoding the four Cav pore-forming domains (CDD output in Additional file 4).

**Table 3:** Summary of putative splice variants detected in long-read RNA-seq data

Gene	Splice variants (minimap alignment and FLAIR processing)	Splice variants with ORF ≳2000 AA	Splice variants with 4 complete pore-forming domains
Cacna1e	2,110	238	154

Initial set of Cacna1e splice variants was 2,110 splice variants. Of those 2,110 splice variants, 238 contained an open reading frame of 2,000 amino acids or greater. We further filtered these splice variants to 154 based on whether they contain the four necessary pore-forming domains

Based on the open reading frame and protein domain annotations, we thus prioritized 154 of the 2,110 splice variants. We interpret the remaining 1,956 of splice variants as likely representing either technical error, such as read truncation during library preparation [43] or splicing noise (see Discussion). Across the 154 prioritized splice variants, we observed 54 exons in total with each splice variant having an average of 44 exons. While all 154 splice variants had four pore-forming domains, we noted that 139 had sequences that encoded for a GPHH domain and 148 had sequences that encoded for an IQ domain.

### Expression profiles of *Cacna1e* splice variants

In the set of 154 putative *Cacna1e* splice variants (Additional file 5), we detected the rbE-II splice variant characterized by Soong and colleagues [44]. None of the transcripts in our data matched the transcripts reported in Ensembl v98, including the sole Ensembl *Cacna1e* transcript that codes with an ORF over 2,000 amino acids (ENSRNOT00000003928). This remained the case even after inspecting the MinION data at the raw read level to ensure that data relevant to this transcript had not been filtered out by our processing pipeline.

**Fig. 2:**
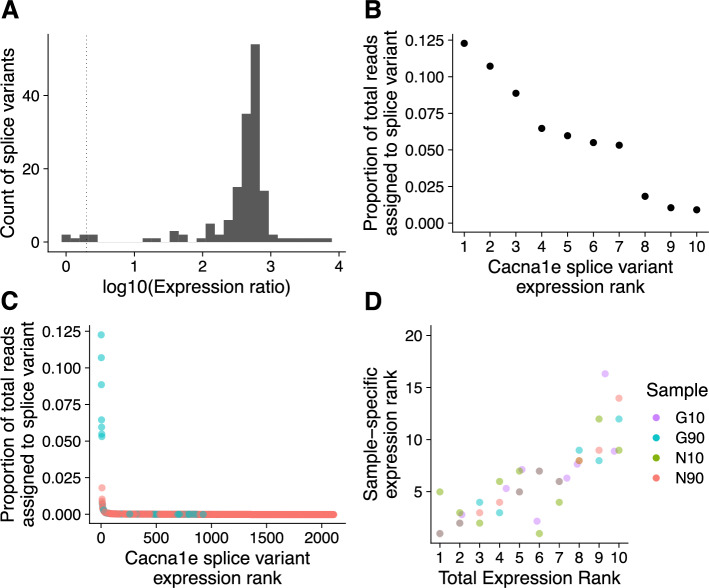
Highly expressed splice variants account for 12.5% of Cacna1e gene expression. A) *Four splice variants have an expression ratio of less than 2.* Each splice variant’s expression ratio is calculated by the most expressed splice variant’s expression divided by the splice variant’s expression. The dotted Line indicates where the expression ratio would equal 2. B) *Top 10 most expressed Cacna1e splice variants pooled across all samples.* The X-axis shows the rank of the splice variant based on expression. The Y-axis shows the proportion of the splice variant relative to Cacna1e’s total expression. C) *Contribution of all 2,110 Cacna1e splice variants to the gene’s overall expression.* The X-axis shows the rank of the splice variant based on expression. The Y-axis shows the proportion of the splice variant relative to Cacna1e’s total expression. Of the 2,110 variants, the 154 (cyan) have 4 annotated pore-forming domains and account for 61% of Cacna1e’s expression. We did not predict the remaining splice variants to encode for all four pore-forming domains (red) D) *Cacna1e splice variant expression level ranks in each sample*. X-axis show expression rank for splice variant summed across all 4 sample, while Y-axis shows the splice variant expression rank for each sample. Sample is indicated by color. G10 is Genetic Absence Epilepsy Rat from Strasbourg at 10 days, G90 is Genetic Absence Epilepsy Rat from Strasbourg at 90 days, N10 is non-epileptic control at 10 days and N90 is non-epileptic control at 90 days. Example: the splice variant at expression rank 1 summed across all 4 sample is the most expressed splice variant G10 and N10. However, for samples G90 and N90, that same splice variant is at rank 4 and 5 respectively

Using the number of reads mapped to each splice variant as a proxy for splice variant expression, we observed that the top four splice variants expressed at similar levels (Fig. 2). These four splice variants each had an expression ratio of less than 2, which indicated that the rank 1 (most expressed) splice variant was double the expression of all but three splice variants (Fig. 2A). The rank 1 *Cacna1e* splice variant matches the splice variant structure and protein sequence of Soong et al. [44]. We mapped 498,616 reads to this most abundantly expressed splice variant, while the second most abundantly expressed splice variant had 435,458 reads. The top three splice variants contributed similarly to the total Cacna1e expression among the 4 samples: 12.2%, 10.7%, 8.8% and 6.5% (Fig. 2B and C). However, when stratifying number of reads per splice variant by sample, we observed that the rbE-II Soong et al. splice variant is the most abundant splice variant in the GAERS90 and NEC90 thalamus samples, and fifth most abundant in GAERS10 and NEC10 (Fig. 2D).

**Fig. 3:**
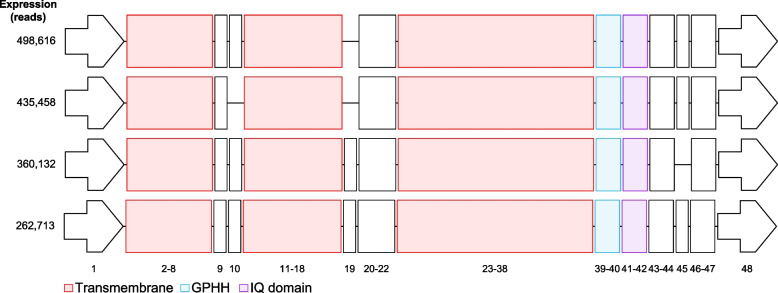
Structure of top 4 most abundantly expressed splice variants. Red indicates a pore-forming domain annotation from CDD (Ion_trans – pfam00520). Blue and purple are calmodulin-binding domain annotation from CDD (GPHH – pfam16905 and Ca_chan_IQ – pfam08763, respectively). Along the side are the number of reads for each splice variant, and long the bottom are the exon numbers. A similar visualization for all 154 splice variants that contained 4 pore-forming domains is provided in Additional File 7

To assess the potential functional diversity of Cacna1e splice variants, we downloaded the protein sequence for Ensembl *Cacna1e* (ENSRNOP00000003928) and rbE-II (1993) then annotated protein domains as a guide for analyzing the thalamic *Cacna1e* splice variants (Fig. 3). Both sequences contained the four pore-forming domains necessary for voltage sensitivity and calcium influx. Furthermore, the Ensembl sequence and rbE-II sequence contained the GPHH and IQ domains (GPHH – pfam16905 and Ca_chan_IQ – pfam08763 respectively) necessary for Ca-calmodulin regulation of channel activity [45].

### *Cacna1e* splice variants contain a conserved cassette exon 19

**Fig. 4:**
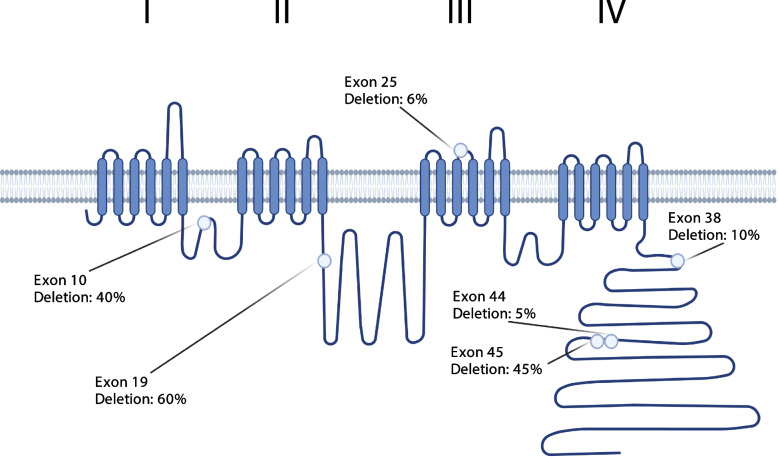
Location of most common splicing events on Cacna1e structure. Here we show where each of these six cassette exons would likely impact the channel, and the percentage of reads that do not contain the exon. Figure created with Biorender.com

We further prioritized the 154 *Cacna1e* splice variants based upon our splice variant-level conservation and expression annotations. We detected 31 cassette exons in the 154 Cacan1e splice variants and show where the six most common skipped exons are located relative to channel structure (Fig. 4). The top three most commonly occurring cassette exons in *Cacna1e* splice variants were: skipping of exon 10 (40% of reads), insertion of exon 19 (40% of reads), and skipping of exon 45 (45% of reads).

**Fig. 5:**
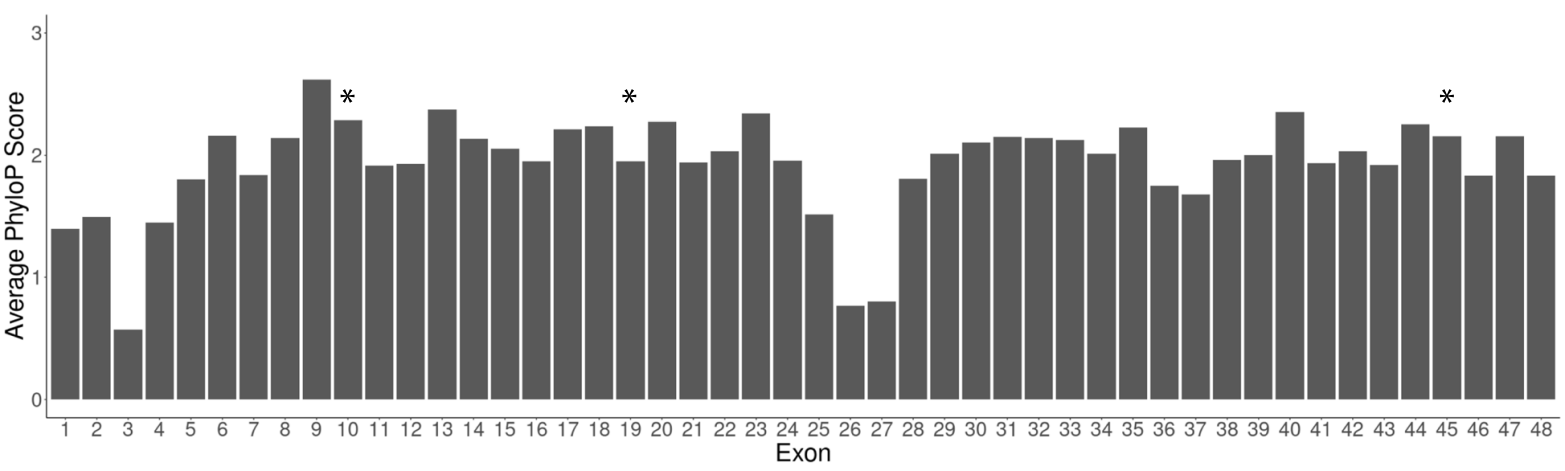
Most Cacna1e exons are well-conserved throughout the vertebrate phylogeny. The UCSC genome browser provides base-specific PhyloP scores. For each exon of Cacna1e, we annotated the average PhyloP score. Our analysis focused on prioritized splice variants with cassette exons 10, 19 and 45 (asterisk)

The protein sequences of exons 10, 19 and 45 are all highly conserved with a PhyloP score over 1.3 (Fig. 5; PhastCons scores displayed in Additional Fig. 1). To determine whether exon skipping is an inherent aspect of function of Cacna1e or due to splicing errors. We investigated whether the exclusion of these exons was conserved; that is, whether transcripts lacking these exons are present in other species. We performed a BLAST analysis to check whether the protein sequences for each exon together with their flanking exons (i.e. exons 9–10-11, exons 18–19-20, and exons 44–45-46) were present in transcribed sequences in other species. We found evidence of conservation for exon 19 and exon 45 within the jawed-vertebrate phylogeny (fish, rodents and human), but only evidence of conservation for exon 10 skipping within the rodent phylogeny. However, the splicing of exons 10, 19 and 44 were all present in the human GTEx long-read RNA-seq dataset, suggesting that these were conserved events between humans and rats. These are consistent with the potential for exon skipping having a conserved function, although as we discuss below the interpretation is not straightforward due to the potential for conservation artefacts.

### Novel *Cacna1e* splicing events

**Fig. 6:**
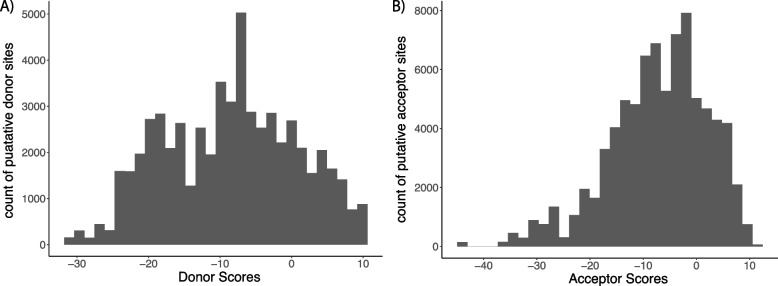
Distribution of MaxEntScores for predicted donor and acceptor sites across 154 putative *Cacna1e* splice variants. **A** Donor scores for sets 9-mers found across the *Cacna1e* splice variants. **B** Acceptor scores for sets of 23-mer found across the 154 putative Cacna1e splice variants

The disconnect between Ensembl and our data is consistent with our previous observation that approximately one-third of transcripts in genomic databases do not reflect experimental literature [12]. Thus, we sought to determine whether our 154 putative *Cacna1e* splice variants would impact computational analyses of the *Cacna1e* gene. Splicing mutations in *Cacna1e* have been implicated in neurological disorders, including epilepsy and pain syndromes, making accurate annotation of its splice variants critical for understanding disease mechanisms. A MaxEntScan [46] analysis comparing splice donor and acceptor sites in our *Cacna1e* splice variants (Fig. 6; Additional File 6) versus the Ensembl-annotated variant revealed that our transcripts had higher median acceptor (−7.99 vs −8.37) and donor scores (−6.25 vs −8.56). This suggests that the alternative splicing patterns captured in our dataset may be influenced by regulatory elements absent from existing annotations and could provide new insights into the functional consequences of *Cacna1e* splicing in disease contexts.

The 154 *Cacna1e* splice variants that had the translation potential to encode functional VGCCs contained a total of 31 splicing events (in various combinations), only six of which had previously been described in the Literature. In total, these splicing events affect less than 11% of the 154 splice variants (17 splice variants), and the splice variants that contained these splicing events accounted for less than 10% of the total reads. We investigated whether there was conservation of these novel splicing events and whether there was any impact to the protein domains. Sixteen of the splicing events involve exons in a pore-forming domain; however, they did not affect the CDD annotation for the pore-forming domain. Two splicing events are the cassette exons encoding for the GPHH and IQ domain. The seven remaining splicing events are exons in the N- or C- termini, or in the cytoplasmic linker regions.

One subclass of variants (14/153) is predicted to result in the lack of an intact calmodulin-binding domain. The most expressed of these had a total of 292 reads and ranked the 350th most abundant splice variant of the 2,110 *Cacna1e* splice variants. The most expressed splice variant lacking a complete calmodulin-binding domain contributed to ~ 0.02% of *Cacna1e*’s total thalamic expression (832 reads). Notably, many of these splicing events lacking calmodulin binding sites have been identified in other species: one is conserved across jawed vertebrates, nine conserved within mammals, and two conserved only within a rodent phylogeny (full results in Additional File 8). Given the low estimated expression levels of these 14 splice variants, additional work would be needed to establish their functional relevance.

## Discussion

Here, we describe the splicing repertoire for the voltage-gated calcium channel (VGCC) gene *Cacan1e* using novel targeted long-read RNA-seq data from the rat thalamus. We computationally prioritized transcripts in terms of their potential to expand the functional diversity of *Cacna1e* channels. This study represents a necessary step towards bridging the gap between claims that alternative splicing vastly increases the functional diversity of VGCC genes and the evidence-based reality of “noisy” splicing. Though we focused on a specific gene, the approaches employed here to computationally assess the biological relevance of splice variants would be applicable to many genes, helping to bridge the gap between raw measures of transcriptional diversity to estimates of functional diversity.

While we detected 2,110 different splice variants for *Cacna1e*, our analyses suggest that the large majority of these are unlikely to be biologically relevant. In particular, 154 (7%) are predicted to encode functional Cav2.3-subunit proteins. Of the remaining 93%, 1,273/1,965 (~ 68% of the total) possess an open reading frame of at least 30 amino acids. The most expressed of these 1,965 splice variants accounts for ~ 1.8% of the total measured *Cacna1e* expression (Fig. 2C). While many Nanopore-driven sequencing studies have reported expression values by raw reads, we were curious whether library size normalization would influence prioritization, thus we quantified the data in terms of transcripts per million reads (TPM; values provided in Additional File 2). The raw counts positively correlated with the TPM values of each *Cacna1e* splice variant (Spearman’s *r* = 0.999), and the top ten most expressed splice variants remained at the same rank. The use of TPM values included only two additional splice variants over and above the four splice variants prioritized with an expression ratio of less than 2.0.

The observation of 1,965 splice variants that do not likely encode functional products agrees with extensive evidence that RNA splicing is imprecise, such that “biochemical noise” is produced and can be captured by modern sensitive molecular biology methods even when present at low levels [11, 14, 47–52]. In line with previous literature exploring transcriptional noise, we hypothesize that the vast majority of detected *Cacna1e* transcripts are likely nonfunctional based on evaluation of multiple lines of evidence: conservation of these transcripts using BLAST homology searches and the GTEx database, amino acid length, predicted protein domains and expression levels. Importantly, since the core function of the *CACNA1E* protein relies on the formation of a voltage-gated calcium channel pore, it would be difficult to argue that a transcript is functional if it does not encode the critical pore-forming domains. While we cannot formally exclude the possibility that some of the non-channel-encoding variants have a function – or that we missed rare but functional isoforms due to technical artifacts or the expression threshold – follow-up studies should prioritize splice variants for which the most plausible case for functionality can be made.

A further distinction of interest is among splice variants that have *different* and required functions from one another, what we call functionally distinct splice isoforms or FDSIs. Computational analyses cannot establish FDSIs, but we applied methods that provide a ranked prioritization. While we detected 154 “full-length” splice variants for *Cacna1e*, evidence from domain analysis, expression and homology focused our attention on a subset of four splice variants involving the skipping of exons 10, 19 and 45 in various combinations. We believe the functional consequences of these three exons are worthy of follow-up. Of note, standard reference-based transcriptome analysis tools are unlikely to predict the four prioritized splice variants. While Ensembl v98’s annotation of exons 10, 19, and 45 individually provides orthogonal evidence that our computational pipeline recovers biologically real transcripts rather than spurious predictions, none of the Ensembl *Cacna1e* transcript models match any of the prioritized isoforms. Similarly, only a single NCBI Cacna1e transcript corresponds to one of the four prioritized variants (Additional file 9).

We failed to find any Literature discussing the functional effects of exon 10 and our BLAST results mapped to a direct GenBank submission without any associated studies (Human NCBI Reference Sequence: XM_017002244.1). The splicing of exon 10 impacts the linker region between domains I-II, the region of the channel where the β-subunits (Cacnb1 genes) interact with the α1-subunit (Cacna1 genes) to both chaperone the VGCC to the cell membrane and affect channel biophysical parameters [53]. Our interpretation of whether the skipping of exon 10 impacts channel function remains limited without *ex silico* validation, although one hypothesis is the loss of exon 10 changes trafficking of *Cacna1e* calcium channels to the plasma membrane.

Both Williams et al. and Schneider et al. reported the cassette splicing of exon 19 [54, 55]. Williams and colleagues showed that splice variants containing exon 19 altered channel inactivation kinetics, however details of the Schneider et al. study were unavailable. The effects of splice variants that contain exon 19 on whole cell current were subsequently characterized in HEK293 cells and a human pancreatic cell line [19, 56]. Of note, investigating the alternative splicing of a different, but functionally similar VGCC gene, *Cacna1b*, Gray and colleagues reported that *Cacna1b* and *Cacna1e* both possess an alternatively spliced 19th exon [57]. Given the conservation of exon 19 and the location of this exon between domains II and III in both *Cacna1b* and *Cacna1e*, Gray and colleagues hypothesized that the exon has an important regulatory role concerning channel function. RT-PCR showed that transcripts containing exon 19 were more prevalent during fetal development than postpartum, and that expression was distinctly absent in the peripheral nervous system [57]. As such, our results provide additional transcript structural context for exon 19 which will be useful for future follow-up.

We failed to find Literature discussing the splicing out of exon 45, and our BLAST results mapped to direct GenBank submissions without any associated study (Human GenBank: AH009158.2, AL161734.12, NG_050616.1). However, exon 45 has been reported to contain de novo variants causing epileptic encephalopathy [10]. Given this potential role of exon 45 in brain disease and the strong conservation of the exon 45’s cassette splicing, exon 45 may be interesting for follow-up.

We note that our study has several caveats and limitations. Our comparison to previous high-throughput sequencing studies should be considered in the context of the low expression of the *Cacna1e* gene and that the detection of multiple transcripts requires a targeted approach. For example, the long-read RNA-seq dataset from GTEx only detects five *Cacna1e* splice variants. Additionally, though others have used PCR-based strategies to estimate expression level, the nanopore-based strategy employed here to isolate *Cacna1e* splice variants is not necessarily expected to lead to accurate expression level estimates for different splice variants, thus relative abundance estimates should be viewed with some caution [24]. Further, we specifically studied the thalamus and the splice variants in other brain regions or tissues could be different. Additionally, with only one sample per condition, and the potential for bias in quantification in cDNA samples which were PCR amplified, we were unable to confidently assess differences in splice variant expression in the GAERS and NEC strains. As such, we minimized this limitation by identifying splice variants found across all four conditions. However, ONT sequencing platforms are continuously improving in terms of efficiency, cost, and accuracy. Moving forward, possible enhancements to the current approach could be accomplished through recent advancements including R10.4.1 flow cells and sample multiplexing with the Rapid Barcoding Kit 96. Finally, while we used conservation of exon skipping events as a way to evaluate the potential for functional relevance, there is an important caveat in that splicing is known to be imprecise in all species [51, 58]. In this regard, we cannot exclude the possibility that observation of an exon skipping event in another species transcriptome is simply the result of chance capture of an “erroneous” isoform. This is made clearer by the observation of “conserved” splicing events that would result in a non-functional channel. Given these limitations, we view our study as prioritizing a set of putative FDSIs for *Cacna1e*, with further high-throughput and low-throughput investigations required to establish their roles and functional importance.

## Conclusions

Our work with *Cacna1e* demonstrates the importance of providing a gene-specific splicing profile using targeted long-read RNA sequencing. We provide transcript structures for 2,110 *Canca1e* splice variants previously missing in genomic databases. Furthermore, our prioritization using splice variant expression and conservation helps point the field towards potentially functionally interesting and relevant splice variants. For *Cacna1e*, the prioritization using expression and conservation provided a mixture of splice variants that have been functionally investigated in the literature (e.g. splice variants containing exon 19) and splice variants without any literature validation (e.g. splice variants containing exon 10). The splice variants detected will provide a foundation for future research into VGCCs for both basic research and therapeutic studies.

## Supplementary Information

Additional file 1. FLAIR generated FASTA file of all Cacna1e splice variants.

Additional file 2. FLAIR generated counts table for all Canca1e splice variants.

Additional file 3. Transdecoder predicted amino acid sequences for all splice variants.

Additional file 4. All Cacna1e splice variants with a predicted CDD domain.

Additional file 5. 154 Cacna1e splice variants with 4 pore-forming domains (predicted by CDD). This file can be used to subset from Additional File 3 or as an input for IsoViz, our isoform visualization tool.

Additional file 6. Donor and acceptor scores using MaxEntScan.

Additional file 7. Full BLAST results of Cacna1e splice variants.

Additional file 8. Visualization of all 154 splice variants predicted to have 4 pore-forming domains.

Additional file 9. Multiple sequence alignment between splice variants identified in this study and *Cacna1e* splice variants from NCBI.

## Data Availability

Raw sequence data are available in GEO under accession GSE295903.
